# The advantages and disadvantages of altruistic and commercial surrogacy in India

**DOI:** 10.1186/s13010-023-00130-y

**Published:** 2023-07-07

**Authors:** Yuri Hibino

**Affiliations:** College of Philosophy in Interdisciplinary Sciences, Kanazawa University, Kakuma-Machi, Kanazawa City, 920-1192 Japan

**Keywords:** India, Commercial surrogacy, Altruistic surrogacy, Exploitation, Regulation

## Abstract

**Background:**

Comprehensive commercial surrogacy became legal in India in 2002, and many foreigners, including individuals and same-sex couples, sought Indian surrogacy services due to their affordability. Numerous scandals resulted, with increasing calls for the government to eliminate the exploitation of women in lower social strata. In 2015, the Indian government decided to exclude foreign clients and commercial surrogacy remained legal for local Indian couples only. Furthermore, to eliminate exploitation, the concept of altruistic surrogacy was introduced in 2016. In 2020, some restrictions within altruistic surrogacy practice were removed. Controversy remains, however, in various sectors, not least because surrogacy is a relatively new concept in India. In this paper, the advantages and disadvantages of both altruistic and commercial surrogacy in the Indian context are considered, and more appropriate policy concerning surrogacy practices is suggested.

**Methods:**

This paper is based on fieldwork conducted in India from 2010 to 2018. Interview surveys were conducted among doctors, policy makers, activists, former surrogates, and brokers. Government documents and media reports were also important sources.

**Results:**

Surrogacy for commercial purposes began in India in 2002, and stakeholders within the commercial surrogacy industry became well established. It was found that such stakeholders were strongly opposed to altruistic surrogacy as introduced in 2016. It was also found that women in lower social strata still sought some form of financial compensation from their reproductive labor. Controversies surrounding altruistic surrogacy continue within Indian society.

**Conclusion:**

Policies and practices aimed at eliminating exploitive need to consider the Indian context carefully. Any surrogacy practice might potentially be exploitive, and the distinction between commercial and altruistic surrogacy is too simplistic to be useful, with more nuanced understanding required. It is of critical importance that investigation continues on how to eliminate the exploitation of Indian surrogate mothers throughout the process, regardless of monetary compensation. The entire surrogacy process should be managed with sensitivity, particularly in relation to the well-being of the mother and child.

## Background

### Commercial surrogacy practice and the emergence of critics against exploitation

India had been well known as a surrogacy hub for foreign clients. Medical tourism policy had been promoted in India based on the idea of trickle-down economics. Women from lower socio-economic strata were an abundant source of surrogate mothers in what became a lucrative business, as costs remained low – about one-fourth the price of surrogacy in the United States – and commercial surrogacy in India became a niche market.

India’s first child resulting from vitro fertilization (IVF) was born in 1986 in Mumbai. In India, advances in reproductive technologies have been linked with nationalistic and cultural ideologies. An anecdote from Hindu religion [1], for example, has been considered to involve assisted reproductive technology including a third party [2], so the use of advanced technologies involving a third-party woman’s body to help an infertile couple has therefore appeared legitimate. Motherhood is glorified in India and infertile couples (especially the women in such couples) are often stigmatized; local people, therefore, tend to sympathize with what they see as the plight of childless couples, irrespective of their country of origin, and may be prepared to help where possible. Furthermore, within a certain religious context, helping infertile couples might also provide good karma for surrogates.

Following a High Court decision allowing commercial surrogacy in 2002, the market grew steadily. The Confederation of Indian Industry predicts that surrogacy generates US$2.3 billion annually [3], with many IVF clinics offering surrogacy programs throughout India. In 2005, the Indian Council of Medical Research (ICMR) issued guidelines on this issue [4], and the government launched related legislative measures. The Assisted Reproductive Technology (ART) Bill was drafted in 2008 and revised in 2010 and 2014 [5]; however, the ART Bill was never enacted. Both the ICMR’s guidelines and the ART Bill made provisions granting custody to the intended parents, including foreigners, with the clear intent of promoting surrogacy tourism.

In July 2012, however, the Indian government suddenly reversed its policy and introduced regulations restricting medical visas to eliminate problems occurring in relation to international clients [6]. From 2002, commercial surrogacy had become common practice throughout India and, as the clientele expanded, several negative incidents, including the death of egg donors [7] and surrogate mothers [8–12], visa troubles affecting a child born from surrogacy [13–15], and the abandonment of surrogate children by clients [16], came to light [17] and created controversy. The concerns voiced regarding the exploitation of women became louder and more persistent [18–20].

As a result of the new regulations, individual foreign clients, gay couples, and those whose countries of origin prohibited surrogacy were no longer permitted to seek surrogacy services in India. In November 2015, all medical visas related to surrogacy services were suspended [21] and, from that time onward, foreigners could no longer visit India for surrogacy purposes.

### Introduction of the altruistic surrogacy concept in India

Following the decision to exclude foreigners from India’s surrogacy market in November 2015, the “Surrogacy (Regulation) Bill 2016” that aimed to regulate the surrogacy market was prepared and introduced in the Lok Sabha (the lower house of India’s parliament) in November 2016. In 2017, the bill was referred to the Parliamentary Standing Committee on Health and Family Welfare, which held a series of meetings and tabled a report on the bill [22].

To reduce exploitation of poor and uneducated local women, the Surrogacy (Regulation) Bill 2016 prohibited commercial surrogacy and permitted only ‘altruistic’ surrogacy within family groups. In the “altruistic” model, surrogates cannot receive monetary compensation for their reproductive labor. The proposed bill allowed altruistic surrogacy under certain conditions, namely, that only Indian couples who had been married for at least 5 years and who had a doctor’s certificate confirming their infertility were allowed to participate, whereas surrogacy arrangement for gay couples, live-in couples, single parents, persons with overseas citizenship of India or person-of-Indian-origin status, and foreigners were prohibited. Furthermore, a woman was to be a surrogate only once and only if she was a close relative of the intended parents, the surrogate mother had to be married and have a biological child of her own, and the intended couple were not to have had any other children, whether biologically or through adoption or through earlier surrogacy.

Any person found to be involved in commercial surrogacy infringing these conditions was punishable with imprisonment and with a fine. Controversy erupted in India among experts concerning this proposed bill. To allow wide-ranging review of the proposed legislation and of its effects on the people involved, the governmental committee responsible for the bill decided to elicit the views of various stakeholders and the general public on the bill through a press release inviting suggestions/views from all concerned people. As a result, an official government report on the Surrogacy (Regulation) Bill 2016 was published in August 2017 [23].

The Surrogacy (Regulation) Bill, 2019 was passed by the Lok Sabha on August 5, 2019. On November 21, 2019, the Rajya Sabha (the upper house of the Indian parliament) adopted a motion to refer the bill to a select committee. The cabinet incorporated all the recommendations of the Rajya Sabha Select Committee before approving the bill. That bill was a reformed version of the draft legislation passed by the Lok Sabha in August 2019. This reformed 2019 Bill was referred again to a select committee, where further changes were made before the cabinet approved what became the Surrogacy (Regulation) Bill 2020. The major changes from the previous proposed 2016 Bill was that the eligibility criteria for being a surrogate mother was widened to include Indian single women, which referred to widows or divorced women aged between 35 and 45 years, in addition to Indian married couples and Indian-origin married couples.

A previous definition of infertility as an inability to conceive after five years of marriage was removed on the ground that this duration was too long for a couple who wanted to have a child.

Within the Surrogacy (Regulation) Bill 2020, the principle of altruistic surrogacy was maintained. However, with the loosening of eligibility criteria concerning a possible surrogate mother, it is likely that surrogacy will become more accessible.

### The dichotomy of commercial surrogacy and altruistic surrogacy

A dichotomy of commercial and altruistic (or non-commercial) surrogacy is widely accepted in various countries where surrogacy is regulated. Altruistic surrogacy is based on an understanding of a ‘gift relationship,’ in which a woman is believed to be motivated by altruism to have a baby for an infertile couple, who are free to reciprocate as they see fit. In contrast, in commercial surrogacy ‘both parties are motivated by personal gain to enter a legally enforceable agreement, which stipulates that the contract mother or “surrogate” is to bear a child of the intending parents in exchange for a fee’ [24].

Globally, there are three principal approaches taken in relation to the practice of surrogacy. The first approach, taken by Germany, France, and China, is that of banning surrogacy completely. The second approach, followed by Israel, Russia, Georgia, and Ukraine, is to make surrogacy completely legal, regardless of whether it is commercial or altruistic. In the U.S., surrogacy is practiced commercially in a few states, proving that it works in a capitalist economy. The third approach is to permit only altruistic surrogacy (and prohibit commercial surrogacy). For altruistic surrogacy, the surrogate mother gives birth to a child without payment, in principle, but she can be remunerated for (and only for) necessary expenses. In many countries including the UK, Australia, Canada, Hong Kong, South Africa, Greece, and Iceland, only altruistic surrogacy is permitted, while commercial surrogacy is prohibited.

The UK provides a standard model of altruistic surrogacy, which has been of some interest in India given the former colonial connection. In the UK, a ‘reasonable’ amount of money can be paid to a surrogate mother, and expenses such as delivery cost, transportation, and lost profit can be remunerated. Indeed, in the UK, reasonable expenses under £12,000 are allowed under the term of altruistic surrogacy, and, according to a report by Surrogacy UK (2015), surrogates typically receive £10,000 − £15,000 (the mean average of compensation paid to surrogates was £10,859) [25], which is not an insignificant amount. This highlights the fact that the line between commercial and altruistic surrogacy is unclear and lacks precise definition. Surrogacy in the UK is regulated by the Surrogacy Agreement Act 1985, which is considered by many to be outdated. Surrogacy law in the UK is to be revised and is currently under discussion. A report by a UK working group on surrogacy law reform proposed that, while surrogacy should remain on an altruistic basis in the UK, the intended parents should become the legal parents without the need for a parental order, meaning they would immediately become parents, and that remuneration for surrogate mothers (i.e. reasonable expenses) should be discussed in greater depth and more precisely defined [23, 26]. Such an approach would appear to involve strengthening the rights of intended parents in relation to the child.

In India, gestational surrogacy is a relatively new concept and the government’s attitude towards surrogacy has changed dramatically within a very short period. Initially, the government was willing to accept international commercial surrogacy as part of the medical tourism industry. They also believed commercial surrogacy would help needy infertile couples around the globe, but, following numerous unexpected scandals, there were increasing public assertions that commercial surrogacy and surrogacy tourism were exploitative of local women [16, 17]. The use of medical visas for surrogacy tourism was stopped, shutting out foreign clients. The government then proposed legislation involving the concept of altruistic surrogacy to eliminate exploitation.

As part of a process investigating how to eliminate exploitation within the practice of surrogacy, this paper considers the advantages and disadvantages of altruistic and commercial surrogacy in India, with the aim of determining exactly what constitutes surrogacy exploitation in the Indian context and to suggest appropriately targeted policy on this issue. First, commercial surrogacy practice within Indian society and the implications for surrogates from lower social strata are examined. Second, the views of local experts and former surrogates concerning altruistic surrogacy and actual practice are reviewed. In the discussion section, the identified advantages and disadvantages of commercial and altruistic surrogacy in the local context are explored in greater depth, and recommendations on how to eliminate exploitation of surrogates are provided.

## Methods

This paper is based on fieldwork conducted in India from 2010 to 2018. During this period, the author visited India eight times and stayed in several areas, including Mumbai, Delhi, Chennai, Anand, and Ahmedabad. Fieldwork regarding views on altruistic surrogacy and the 2016 Surrogacy (Regulation) Bill was conducted in January at Mumbai, and in July and October at Delhi, in 2018. The most recent field survey was conducted in Delhi between 13 and 20 October 2018. People’s views regarding altruistic surrogacy as proposed in the 2016 Surrogacy (Regulation) Bill and current surrogacy practices by local practitioners were also surveyed. Informants at the time of this visit included two policy makers, three non-government organization (NGO) members, one journalist, two doctors, and one surrogate broker who was also a former surrogate.

On-site, face-to-face interviews were supplemented with interviews conducted over Skype when necessary. Interviews were conducted in English or in the local language, as applicable. Interpreters provided assistance. Most interviews were audio-recorded, and interviews in local languages were transcribed and subsequently translated to confirm details. Transcripts are cited in this paper to illustrate the findings. Citations of interview data are presented without identifying information to protect the privacy of participants. Relevant government documents and media reports were also important sources and were analyzed accordingly.

## Results

### Commercial surrogacy practice and its implication for the surrogates

Many IVF clinics have offered commercial surrogacy programs throughout India since 2002. Of these, surrogacy practice in Anand is known for its outstanding and media-friendly features. Although the surrogacy practice undertaken in Anand is perhaps not typical of practices throughout India, it appears to have influenced discourse on commercial surrogacy conducted in India. I visited Anand in 2012, where more than a hundred pregnant women were living in a surrogate hostel offered by the surrogacy clinic for its surrogates. In the surrogate hostel, each woman was provided with a small space around a single bed made of steel. The women were well cared for and monitored under the supervision of the neighboring clinic. Inside the hostel, the women could chat with each other, watch TV, and receive free job training provided by a doctor. Family members were also permitted to visit the surrogates on the weekend. Although supervision of the body of the surrogate during the nine months of pregnancy was exclusively for the benefit of the intended parents, it also provided an ‘alibi’ for the women; through staying nine months in the hostel, they could remain hidden from neighbors who might gossip about them [27]. For local ordinary people, surrogacy was considered a good thing for childless couples, since those couples had the chance to have children. In contrast, the women who delivered the children for those childless couples tended to be stigmatized. A sharp divergence in social attitudes towards aspects of surrogacy was apparent, as has been observed in another study [28].

The practice of commercial surrogacy in Anand and elsewhere in India became increasingly controversial, drawing mixed reactions [29, 30]. Dr. Nayana Patel, who ran the IVF clinic in Anand, had a good reputation locally. Indeed, Anand and its surrounding area received clear financial advantages from surrogacy tourism. The intended parents often had an extended stay while undergoing the IVF procedure at the beginning of the process, and to obtain a visa for the newborn baby after the delivery. These foreign couples and/or individuals tended to spend their money in hotels and in baby goods shops around Anand, which suited local business owners and employees. Other local people, especially those not familiar with the IVF procedure, are unaware that IVF can help women become pregnant without intercourse and appeared to believe that the doctor was helping childless couples to have a child in an immoral way.

One NGO director in Anand noted in an interview that becoming a surrogate mother for childless couples was a much better option than prostitution to earn money, so she would definitely recommend it to poor families. She stated:

Surrogacy is very popular here. The women out here are very independent and bold in their thinking. They do not worry or bother about what society says. They can make decision by themselves. People here place very much importance on commercial dealings. They feel that by doing this she can ensure a good future for their own children. It is their own body, so they can do with it what they wish. I feel it is good work as couples get children and these women also get money. Women here are very much empowered. (Observation of an NGO director in Anand in 2012)

According to Amrita Pande, who conducted a field survey for several months in Anand, the income from commercial surrogacy enabled such families to give their children, including their daughters who tend otherwise not to be educated, a good education and improve their options in the future [25]. In the field survey conducted for this study, the women in the surrogate hostel expressed their gratitude both to their gods and to the doctor for the opportunity to become surrogates. In their terms, they would receive a substantial sum of money after nine months and this money was likely to improve their lives. However, it was apparent that many surrogates staying in the surrogate hostel were pregnant with twin babies, and these expectant mothers expressed considerable fear concerning delivery. Also, they had to prepare for the day when they would relinquish their baby immediately after the delivery. Thus, they faced various and demanding physical and emotion challenges before they could obtain financial recompense.

Foreign media had visited Anand and some journalists had described the surrogacy clinic as a ‘baby factory’. From a certain viewpoint, using the bodies of poor women in this way and housing surrogate mothers in a hostel under the supervision of a clinic could appear inhumane and unjustifiable [28]. The doctor running the IVF clinic in Anand addressed those holding such a viewpoint by arguing that: ‘Through commercial surrogacy, desperate intending parents’ dreams come true. They have a baby, and poor families benefit financially. Surrogacy is a win–win situation’ [31]. Also, the doctor drew attention to the fact that, in the U.S., commercial surrogacy was widely practiced, but that, with regard to Indian women, exploitation was always an issue, which she considered to be unfair.

For Indian women from lower social strata who participated in commercial surrogacy, obtaining money was an important issue and they were clearly motivated to improve their living standards, as indicated in many testimonies cited in media coverages, reports and academic journals [32]. However, the money received has been always been considered adequate. As one person closely involved in the industry in Mumbai stated:

Undoubtedly, 3 lakh [1lakh=100,000 Indian rupees] or 4 lakh is big money for poor families. In Anand, they could buy a very small house because it is in the countryside. However, here in Mumbai, even though you could buy a house, it would be a very poor house located far from the central areas. In the surrogacy business, while the doctors earn a lot of money, surrogate mothers do not. I personally believe the women should receive much more money. Moreover, even if the women receive a certain amount of money, their lives do not change very much. By contrast, the lives of the intended parents change dramatically if they obtain a baby. (Observation from a surrogate broker and former surrogate in Mumbai in 2012)

According to this broker, in many cases, the money was spent in a short time and the situations of these women tended not to change. The money being paid was considered insufficient to allow certain poor families to improve their lives sustainably or to facilitate their joining middle-class society if that were their wish. This broker believed that surrogates were not receiving enough, given the physical and mental hardships involved, and that, therefore, they would have a sense of being exploited even if what they received might be thought as considerable in their terms.

When the Indian government proposed a draft bill on surrogacy that would ban all commercial surrogacy in the country, allowing only close family relatives to become surrogate mothers, Dr Nayana Patel in Anand organized a demonstration in 2015 with the surrogates to protest against the proposed legislation [33]. The doctor claimed that, in banning commercial surrogacy, poor families stood to lose a lucrative income. As altruistic surrogacy within family members comprised only 25 cases of the 1,000 cases that the doctor was dealing with, this meant that, if surrogacy was limited to family members, most needy infertile couples would never be able to access it. Other stakeholders in the commercial surrogacy industry, such as agencies and lawyers in addition to doctors in IVF clinics, expressed strong opposition to the proposed bill, not least because it would damage their businesses.

### Controversies surrounding altruistic surrogacy

As noted regarding the Surrogacy (Regulation) Bill 2016, the governmental committee responsible for reviewing the bill sought input from various stakeholders and the general public. Public hearings concerning the bill were held and a report was published, which noted views claiming that, in various ways, altruistic surrogacy could be considered impractical, draconian, discriminatory, and based on an outmoded patriarchal model [34]. Finding a surrogate among family members is difficult since this model is based on a patriarchal extended familial structure and does not work within a nuclear family commonly residing in an urbanized area. Additionally, others declared surrogacy restricted to family members to be unrealistic and that no one would be willing to be a surrogate mother without adequate payment. While there was understandable opposition from the affected business sectors and from the surrogacy industry such as IVF specialists, other sectors of society not directly affected as well as certain feminists also expressed opposition.

According to one journalist, commercial surrogacy had certain advantages, particularly for poor families:

There have been many successful cases involving commercial surrogacy in Anand. Many poor families lack education and have no well-paying employment, so commercial surrogacy might in fact help to improve their lives. Activists, including feminist groups, claim that exploitation is occurring, but surrogate mothers are not making the same claim. Indeed, for surrogate mothers, money is important, and they tend to say nothing as long as they are well remunerated. Their voices can be easily neglected in this hierarchal society.(Observation from a journalist in Mumbai, January 2018)

Numerous testimonies have been reported articulating a similar standpoint on commercial surrogacy [30]. Also, the surrogate broker and former surrogate in Mumbai stated her complete opposition to altruistic surrogacy and that it should not be introduced into Indian society, claiming:

I never see altruistic surrogacy cases. I think altruistic surrogacy involving relatives only does not work here in India.

Firstly, because childless couples do not like to ask their relatives to become involved in surrogacy. Secondly, because they want to maintain their privacy, whereas they would definitely ask commercial surrogates. Thirdly, concerning poorer women, they could never make such a large amount of money from ordinary work. Indeed, foreigners are more welcome because they pay more and can give additional money as tips. Finally, I think surrogacy is surrogacy and it makes no difference whether it’s commercial or altruistic – I mean, the two are almost the same. If the government prohibits commercial surrogacy, we will pay bribes and continue to do it. (Observation from a surrogate broker and former surrogate in Mumbai, January 2018)

In the report on the bill [20], the chairperson of the government committee on surrogate women pointed out that the government should give poor women an education so they could find well-paying employment, and that earning money though surrogacy should not be a legitimate option [21]. For the Indian government, empowering women is a critical long-term objective. At present, however, poor families must survive day-to-day with little income and confronted with prices that increase yearly. Becoming a surrogate mother is likely to be considered a compelling option in this situation, from which one could infer that some women would still want to become surrogate mothers even if commercial surrogacy was to be completely prohibited. Indeed, as the broker in Mumbai cited formerly stated:

We now have a plan to transfer Indian surrogate mothers to another country where embryo transfer and delivery can happen, as foreigners are prohibited from entering India to procure surrogacy. The plan remains under review because we have to check the laws in potentially favorable countries. Our group member comprises an IVF doctor, a lawyer, and a caretaker and we are meeting next week for further discussion. (Observation from a surrogate broker and former surrogate in Mumbai, January 2018)

For lower-class Indian families, the provision of pregnancy and childbirth services for others as a means of earning money had become well established and was well known. Even with strict legislation, loopholes are likely to be found. For example, after international surrogacy was effectively banned in 2015 in Thailand, transferring surrogate mothers to neighboring countries has been reported [35], which greatly increases the risks in such cases as those countries do not provide protection nor does local law protect local women who provide surrogacy outside of the country, rendering such women especially vulnerable [17]. Indeed, some scholars have pointed out that, with stricter regulations, a black market is likely to emerge and the situation for surrogate mothers could become even more unjust [21, 34, 36].

## Discussion

### Why is the altruistic surrogacy model impractical in contemporary India?

To address serious concerns regarding commercial surrogacy and to eliminate exploitation of surrogate mothers within commercial surrogacy in India, the concept of altruistic surrogacy was first formally introduced within India through the Surrogacy (Regulation) Bill 2016, which led to the exclusion of foreign clients for surrogacy services. However, in further revisions, some restrictions in relation to altruistic surrogacy were removed in early 2020. The Surrogacy (Regulation) Bill 2016 prohibited payments for surrogates and limited being a surrogate mother to close relatives of the intended parents. Objections were swiftly raised, mainly from stakeholders in the business sector, who claimed that altruistic surrogacy was impractical, draconian, discriminatory, and based on a patriarchal model [37]. Two important issues in particular emerged, namely, the constraining of surrogacy to family members and the matter of compensation for the surrogates.

Given the stigma surrounding infertility, there is a strong motivation among couples to keep their situation secret. Many couples would prefer that the surrogate be an unknown woman, rather than a family member. Indeed, according to the broker interviewed in Mumbai, couples generally prefer commercial surrogacy for this very reason. It is possible that some couples who hold traditional beliefs may feel uncomfortable seeking a surrogate mother from lower social strata, but because the concept of commercial surrogacy had become well established in India, it offered wider opportunities. Commercial surrogacy had also gained increasing support from other stakeholders, such as the intended parents.

The altruistic surrogacy model proposed in the Surrogacy (Regulation) Bill 2016 was clearly based on India’s traditional social (patriarchal) system, where an extended family is the norm and reciprocal relationships between family members are presumed to be paramount. Society is rapidly changing, however, especially in urban areas. People can no longer rely on familial ties to the same extent and, for those urbanized people, monetary remuneration might be a more appropriate approach than familial reciprocity, which is based on the premise of an existing long-term relationship.

On the other hand, restricting altruistic surrogacy to close relatives has advantages for both intended parents and surrogates, because when a surrogate mother is a family member, the intended parents know her well and are likely to feel more secure. Moreover, the similarity of lifestyle between the intended parents and the surrogate mother might also be a great advantage, as Indian society is stratified by religion and caste. As for the surrogate mother, she is likely to be well cared for and, after the delivery, she can have a relationship with the intended parents and the child. However, if surrogacy is limited to family members, the family may apply pressure within the familial hierarchy, which could undermine a woman’s ability to make choices for herself. Such an outcome can be problematic, and for that reason it was argued by many that restricting altruistic surrogacy to relatives should not be confirmed in law.

Traditionally, people in Indian society generally sympathize with heterosexual couples and women facing involuntary childlessness (however, within the Indian system of values, there is no social approval for single males or homosexual couples raising a child). Given this context, surrogacy would appear to an attractive option but with modifications to the altruistic surrogacy model needed to enhance its feasibility. After social debate, several restrictions were removed and the altruistic surrogacy model was made more flexible in the Surrogacy (Regulation) Bill 2020.

### A modification of the dichotomy between commercial and altruistic surrogacy in the Indian context

From 2002, international commercial surrogacy in India involving non-Indians developed steadily until it was made illegal in 2015. However, until the Surrogacy (Regulation) Bill 2020 is enforced, commercial surrogacy for local parties is not illegal. Given this long exposure to commercial surrogacy, its negative aspects have become well known. However, women from poor local families do not want to lose the financial benefits they stand to gain from commercial surrogacy, as indicated through the testimonies presented in this paper and in other publications [30]. Their voices should be heard when formulating surrogacy legislation in India, especially as their views might otherwise be easily neglected within the hierarchal structures of Indian society.

Based on these findings of this study, a modification in the dichotomy between commercial and altruistic surrogacy is recommended. These distinctions were first introduced in the UK and then followed by other Western countries. In a certain understanding, commercial surrogacy has been regarded as exploiting surrogate mothers, whereas altruistic surrogacy does not. It has been claimed that a woman’s free will and autonomy can be undermined if she has a strong financial motivation. Based on such a viewpoint, some countries have removed the possibility of women becoming surrogate mothers for financial gain. However, that approach focuses too much on whether money is being paid to the surrogate, whereas exploitation is a substantially more complex issue, which renders the distinction between commercial and altruistic surrogacy ultimately naïve and simplistic [38]. In the case of India, exploitation is not limited to financial issues as indicated, but should be considered in the context of Indian society generally in relation to the situations actually faced by women [39].

More broadly, various scholars are increasingly questioning the portrayal of poor women in third world countries as victims. In western culture, the value of altruism is emphasized, particularly as an appropriate form of motivation, and this tendency is reflected in the preference for altruistic surrogacy. Surrogate mothers in India have been portrayed as victims of poverty who have no other choice but to participate in surrogacy for financial gain [40]. Such an understanding appears to involve cultural bias. Whether surrogacy is considered commercial or altruistic, and how such distinctions are viewed, involves culturally based assumptions to some extent, with varying understandings and evaluations possible depending on the cultural context. Indeed, in Australia, where only altruistic surrogacy had been legally accepted, a paid/compensated surrogacy model has been introduced [41]. In such alternative models, informed consent and the autonomy of surrogate mothers are highlighted.

As illustrated through the testimony of local informants, banning payment for surrogacy does not consider local realities in which many poor families are falling behind in a rapidly changing society and suffering economic difficulties. If surrogacy is not tightly restricted, Indian women will not be tempted to go abroad to become surrogate mothers, which is an extremely high-risk undertaking. It has been contended that the Indian government does not need to focus so much on commercial surrogacy practice given that, while commercial surrogacy can be exploitative, so too can altruistic surrogacy be exploitative for local women [40, 42]. The 2020 Bill does not protect the rights of surrogate mothers. Moreover, it deprives poor women of the chance to become surrogate mothers and earn money in that way [43]. Given this context, it would appear more reasonable for the government, in seeking to reduce exploitation, to focus more on improving the autonomy, bargaining power, and empowerment of the women involved in surrogacy practices.

The form of surrogacy proposed in the most recent bill shows a clear lack of advocacy for the surrogate mothers it is intended to aid, which is striking given that protection of the rights of surrogate mothers and of the children has been considered of paramount importance. Certain measures are required. For instance, protection of reproductive health is important. Moreover, limiting the number of embryos transferred is a significant consideration. In India, multiple embryo transfers are common to increase the success rate of pregnancy [28, 44], but interventions to facilitate the intended parents’ preferences must be made illegal. In commercial surrogacy [16], compensation is paid to surrogates after they part with the baby. If she terminates the pregnancy, she will not be compensated reasonably. Moreover, she must give up the baby after the delivery to receive money. This practice is unacceptable. While considering commercial surrogacy, such practices could be eliminated via thorough inspections of the surrogacy process, and any allegations made by the surrogate mother whether before, during or after the surrogacy process, could be investigated carefully. The Bill says that The Surrogacy Board will be established in order to collect precise data, but it should not only collect statistics, but help resolve issues or claims arising from malpractice. Moreover, grass-root organizations for surrogate mothers should be encouraged with the help of a government body, given that many Indian women have experience with surrogacy and thus could advocate for prospective surrogate mothers. Such activity would contribute towards the empowerment of poor women. Finally, the right of surrogate mothers to know their children born through surrogacy should be advocated for. Having the possibility of contact with their children and with the intended parents after delivery has been reported as being important for surrogates [25, 45]. Allowing such contact may be a further motivation to become a surrogate mother.

However, in commercial surrogacy, contact tends not be allowed after delivery. In general, surrogate mothers are well taken care of during pregnancy because they are bearing the child of the intended parents. However, after delivery, the surrogate mother is of no further interest as her services are no longer required. After compensation, the surrogate cedes her rights to the child she bore. Nonetheless, refusing to allow contact might have a detrimental effect not only on the surrogate mother but also on the child. The surrogate mother is important because she is a biological mother of the child. Anecdotally, the right of children to know their surrogate mothers is a significant factor, but it was never discussed in government reports nor among other specialists in this sector. Children’s rights should be discussed and be reflected in legislation.

### Recommendations to eliminate exploitation

To eliminate the exploitation of surrogate mothers, the entire process needs to be thoroughly monitored, regardless of whether it is commercial or altruistic [47]. It is of primary importance that the surrogate mother has complete autonomy throughout the whole process, because it is her body that is involved. Surrogacy should be explained fully to the patient to gain their informed consent. For example, pregnancy through IVF carries an elevated risk of preterm labor, maternal and fetal complications, and risks of abnormal placentation. The mode of delivery, if elective cesarean section, bears a greater risk than a vaginal delivery during and after the delivery. Moreover, surrogates should be made aware of the rare intrapartum and postpartum complications by the delivering physician and hospital.

Commercial surrogacy can be practiced as the relevant law has not been enacted up to 2021. Compensation for surrogates is lower since overseas patients cannot visit India anymore. At present, surrogacy is riskier for poor local women. Therefore, a regulatory law for surrogacy must be enacted. Any surrogacy practice might potentially be exploitative, and the distinction between commercial and altruistic surrogacy is too simplistic to be useful and should be discarded [41]. More important is the continued investigation of ways to eliminate the exploitation of surrogate mothers throughout the process, regardless of surrogacy type. Government-funded advocates should check contracts in detail and, after the process begins, any elements found to be disadvantageous to the surrogate mother throughout the process should be eliminated. The entire surrogacy process should be managed with sensitivity to ensure the well-being of the mother and child.

## Conclusion

Based on the findings of this study, a modification in the dichotomy between commercial and altruistic surrogacy is recommended. Any surrogacy practice might potentially be exploitative, and to eliminate the exploitation of surrogate mothers, the entire process needs to be thoroughly monitored, regardless of whether it is commercial or altruistic. Given the Indian context, it would appear more reasonable for the government, in seeking to reduce exploitation, to focus more on improving the autonomy, bargaining power, and empowerment of the women involved in surrogacy practices.

## Data Availability

Data sharing not applicable to this article as there are no consents from informants.
